# Androgen-deprivation therapy and cognitive decline in the NEON-PC prospective study during the COVID-19 pandemic

**DOI:** 10.1016/j.esmoop.2022.100448

**Published:** 2022-03-07

**Authors:** N. Araújo, A. Costa, L. Lopes-Conceição, A. Ferreira, F. Carneiro, J. Oliveira, I. Braga, S. Morais, L. Pacheco-Figueiredo, L. Ruano, V.T. Cruz, S. Pereira, N. Lunet

**Affiliations:** 1 EPIUnit – Instituto de Saúde Pública, Universidade do Porto, Porto, Portugal; 2 Laboratório Para a Investigação Integrativa e Translacional em Saúde Populacional (ITR), Porto, Portugal; 3 Instituto Português de Oncologia do Porto, Rua Dr. António Bernardino de Almeida, Porto, Portugal; 4 Instituto de Investigação em Ciências da Vida e Saúde, Escola de Medicina da Universidade do Minho, Campus de Gualtar, Braga, Portugal; 5 Departamento de Ciências da Saúde Pública e Forenses e Educação Médica, Faculdade de Medicina da Universidade do Porto, Alameda Professor Hernâni Monteiro, Porto, Portugal

**Keywords:** prostate cancer, neurocognitive disorders, longitudinal studies, hormones, hormone substitutes, hormone antagonists/analogues and derivatives, COVID-19, complications

## Abstract

**Background:**

Androgen-deprivation therapy (ADT) has been associated with cognitive decline, but results are conflicting. This study describes changes in cognitive performance in patients with prostate cancer, according to ADT, during the first year after prostate cancer diagnosis.

**Patients and methods:**

Patients with prostate cancer treated at the Portuguese Institute of Oncology of Porto (*n* = 366) were evaluated with the Montreal Cognitive Assessment (MoCA), before treatment and after 1 year. All baseline evaluations were performed before the coronavirus disease 2019 (COVID-19) pandemic and 69.7% of the 1-year assessments were completed after the first lockdown. Cognitive decline was defined as the decrease in MoCA from baseline to the 1-year evaluation below 1.5 standard deviations of the distribution of changes in the whole cohort. Participants scoring below age- and education-specific normative reference values in the MoCA were considered to have cognitive impairment. Age- and education-adjusted odds ratios (aORs) were computed for the association between ADT and cognitive outcomes.

**Results:**

Mean MoCA scores increased from baseline to the 1-year evaluation (22.3 versus 22.8, *P* < 0.001). Cognitive decline was more frequent in the ADT group, and even more after the onset of the COVID-19 pandemic (aOR 6.81 versus 1.93, *P* for interaction = 0.233). The 1-year cumulative incidence of cognitive impairment was 6.9% (9.1% before and 3.7% after the pandemic onset), which was higher among patients receiving ADT, but only after the pandemic (aOR 5.53 versus 0.49, *P* for interaction = 0.044).

**Conclusions:**

ADT was associated with worse cognitive performance of patients with prostate cancer, mostly among those evaluated after the first COVID-19 lockdown.

## Highlights

• On average, cognitive performance of patients with prostate cancer improved during the first year of treatment.
• Nearly half of the patients with cognitive impairment before treatment improved after 1 year.
• Patients treated with ADT were more likely to present incident cognitive impairment at 1 year.
• The COVID-19 pandemic may have worsened the effect of ADT in the cognitive performance of patients with prostate cancer.

## Introduction

With nearly 5 million 5-year prevalent cases estimated in 2020, patients with prostate cancer represent the largest population of male cancer survivors worldwide.^1^ Nearly half of these patients may have been submitted to Androgen-deprivation therapy (ADT) during the course of the disease.^2^ ADT is used in clinically localized prostate cancer to complement radical radiotherapy; in regional disease (lymph nodes affected), alone or associated with radiotherapy; in metastatic disease; and in persistent or recurrent disease after radical prostatectomy or radiotherapy.^3^ However, ADT has been associated with several adverse effects, including cognitive decline and dementia. Most studies on cognitive decline were small and yielded heterogeneous results, and have been summarized in a meta-analysis that showed an association between ADT and a decline in visuomotor tasks.^4^ More recently, retrospective studies based on large health records, claims, and other administrative electronic databases found conflicting results on the association between ADT and dementia.^5^, ^6^, ^7^, ^8^, ^9^ In the available prospective studies, an accurate assessment of the potential effect of ADT on cognitive performance was limited by instrument variability, small sample sizes, and short follow-up duration.^10^ Moreover, cognitive outcomes were essentially based on the variation in cognitive performance from a baseline to a follow-up evaluation, and there is no study reporting the incidence of cognitive impairment, defined as a performance below the expected, accounting for age and education.^11^

Therefore, in a cohort evaluated before treatments for prostate cancer and after 1 year, this study aimed to compare the variation in cognitive performance scores and the incidence of cognitive impairment between patients treated with ADT and those who received treatments without ADT. The follow-up period comprises the pre- and post-first lockdown periods due to the coronavirus disease 2019 (COVID-19) pandemic. Because the restrictions to daily life activities imposed to control the pandemic may have contributed to less cognitive stimulation^12^ and worsening of cognitive impairment,^13^ data were also analysed taking into account the possible effects of the pandemic on cognitive performance of patients with prostate cancer.

## Methods

The NEON-PC prospective cohort study was developed at the Portuguese Institute of Oncology of Porto (IPO-Porto), and has been described in detail elsewhere.^14^ In brief, between February 2018 and March 2020, patients recently diagnosed with prostate cancer and proposed for any treatment, including active surveillance, and those with a disease recurrence to be treated with ADT, were considered eligible and consecutively recruited. Illiterate patients and non-Portuguese native speakers were excluded, as well as those with a previous history of chemotherapy, radiotherapy, or ADT, and those with a neurologic or psychiatric condition impairing cognitive performance diagnosed before prostate cancer. Patients were recruited at the end of the multidisciplinary tumour board meeting when the different available options to treat their cancer were proposed.

A total of 486 participants were evaluated at baseline and 366 (75.3%) at the 1-year evaluation. All baseline evaluations were concluded before the COVID-19 pandemic and 69.7% of the 1-year assessments were performed after the first lockdown due to the pandemic. A total of 120 participants were not evaluated at 1 year because their evaluation was postponed due to the pandemic (*n* = 66), or were lost to follow-up, due to refusal to participate (*n* = 36), follow-up at another hospital (*n* = 5), severe hypoacusia precluding the 1-year evaluation (*n* = 1), ADT refusal (*n* = 1), brachytherapy not performed because of diagnosis and treatment with chemotherapy for another primary tumour (*n* = 2), or death (*n* = 7). Those who did not perform the 1-year evaluation had a lower educational level [education in years, median [percentile 25-percentile 75 (P25-P75)]: 4 (4-8) versus 5 (4-10); *P* = 0.013] and had a lower baseline score in the Montreal Cognitive Assessment (MoCA) [mean, standard deviation (SD): 20.6 (4.12) versus 22.4 (3.69); *P* < 0.001]. Participants evaluated at 1 year received treatments including ADT more frequently and underwent brachytherapy less frequently (*P* = 0.006; Table 1).

**Table 1:** Characteristics of the participants evaluated at 1 year

	Participation at 1 year		*P* value
	No *N* = 120	Yes *N* = 366	
Age (years), mean (SD)	68.1 (6.95)	67.8 (7.27)	0.736
Education (years), median (P25-P75)	4 (4-8)	5 (4-10)	0.013
MoCA, mean (SD)	20.6 (4.13)	22.4 (3.69)	<0.001
Cancer stage, *n* (%)			0.001
I	14 (11.7)	20 (5.5)	
II	63 (52.5)	150 (41.0)	
II/III	3 (2.5)	3 (0.8)	
III	28 (23.3)	116 (31.7)	
IV	12 (10.0)	77 (21.0)	
Treatments, *n* (%)			0.006
Active surveillance	8 (6.7)	18 (4.9)	
Brachytherapy	37 (31.1)	52 (14.2)	
RT	13 (10.9)	38 (10.4)	
RP	22 (18.5)	59 (16.1)	
RT + ADT (6 months)	15 (12.6)	35 (9.6)	
RT + ADT (24 months)^a^	16 (13.8)	90 (24.6)	
ADT (incident disease)	4 (3.4)	22 (6.0)	
ADT + chemotherapy	1 (0.8)	12 (3.3)	
ADT (recurrent disease)	6 (5.0)	25 (6.8)	
RT + palliative ADT	0	1 (0.3)	
RP + RT	2 (1.7)	13 (3.6)	
RP + ADT	0	1 (0.3)	

ADT, androgen-deprivation therapy; MoCA, Montreal Cognitive Assessment; P25, percentile 25; P75, percentile 75; RP, radical prostatectomy; RT, radiotherapy; SD, standard deviation.

a Participants were proposed for 24 months of ADT and were still on ADT at the 1-year evaluation.

At baseline and at the 1-year evaluation, the cognitive performance of participants was evaluated with the MoCA. This cognitive test was developed to detect mild cognitive impairment, and demonstrated good sensitivity and specificity. It assesses eight cognitive domains with 12 tasks and its score ranges from 0 to 30, with lower scores indicating worse cognitive performance.^15^ Participants completed the Hospital Anxiety and Depression Scale (HADS), and anxiety and depression subscores ≥11 out of 21 were considered indicative of clinically significant anxiety and depression symptoms, respectively.^16^^,^^17^

Clinical information regarding prognostic cancer stage group and treatments performed was retrieved from medical files. Prognostic cancer stage group, based on the tumour, nodes, metastases classification, Gleason grade, and prostate-specific antigen, was defined according to the AJCC (American Joint Committee on Cancer) TNM system, eighth edition.^18^ Gleason scores were grouped into Gleason grades according to the International Society of Urological Pathology.^19^ This is an observational study and participants were treated according to usual practice at IPO-Porto. First-line drugs used in ADT included goserelin with or without bicalutamide or, in a few cases, degarelix; second-line treatment included abiraterone acetate and enzalutamide. Most patients admitted to IPO-Porto with symptomatic metastatic prostate cancer were prescribed 150 mg bicalutamide per day at the first consultation until the administration of goserelin, to prevent testosterone flare. In these cases, the baseline evaluation was performed approximately 3 weeks after initiating antiandrogen but before the first goserelin administration. Docetaxel was used for chemotherapy.

### Statistical analysis

Patients’ characteristics are described using counts and percentages, means and SD, or medians and P25 and P75.

Based on the mean and SD of age- and education-specific norms,^20^ MoCA *z*-scores and *t*-scores were computed based on the formula (*z*-score × 10) + 50, to obtain a more intelligible score, so that most values are positive and vary from 0 to 100.

Variation in cognitive performance was computed as the difference between MoCA at 1 year and at baseline. Participants with a variation below 1.5 SD of the distribution of changes in the cohort were considered to have cognitive decline.

Participants were considered to have cognitive impairment when scoring in the MoCA, below age- and education-normative reference values (1.5 SD below the mean^20^^,^^21^). Among participants with no cognitive impairment at baseline, those presenting cognitive impairment at the 1-year evaluation were considered to have incident cognitive impairment.

The incidence of cognitive impairment and cognitive decline was compared between the ADT group and the non-ADT group using multivariate logistic regression to estimate odds ratios (ORs) and the corresponding 95% confidence intervals (95% CIs). The ADT group included patients treated with ADT only, those treated with radiotherapy (with or without brachytherapy) and ADT, those treated with ADT and chemotherapy, and those with persistent disease after radical prostatectomy and/or radiotherapy, treated with ADT. Stratified analyses were conducted according to the moment of the 1-year follow-up, and interaction terms computed: before versus after the onset of the pandemic.

## Results

Participants with the 1-year evaluation performed after the pandemic onset were more educated (55.7% versus 50.6% had >5 years of education, *P* = 0.016) but were similar in age and lifestyles. Nearly half never smoked and were practicing the recommended amount of physical activity, nearly 30% had a body mass index <25 kg/m^2^, and half had hypertension (Table 2).

**Table 2:** Characteristics of the participants, according to the period, pre- or post-COVID-19 pandemic onset, of the 1-year evaluation

	Timing of the 1-year evaluation			*P* value
	All	Before the COVID-19 pandemic	After the COVID-19 pandemic onset	
	*n* (%)	*n* (%)	*n* (%)	
Age				0.746
≥68 years (median)	173 (47.3)	71 (48.3)	102 (46.6)	
Education				0.016
≥5 years (median)	185 (50.6)	63 (42.9)	122 (55.7)	
Smoking status				0.425
Never smoker	158 (44.1)	62 (43.4)	96 (44.7)	
Ex-smoker	164 (45.8)	63 (44.1)	101 (47.0)	
Current smoker	36 (10.1)	18 (12.6)	18 (8.4)	
Excessive alcohol consumption^a^	151 (44.8)	64 (47.8)	87 (42.9)	0.376
Recommended physical activity^b^	159 (43.4)	64 (43.5)	95 (43.4)	0.976
Body mass index (kg/m^2^)				0.104
<18.5	1 (0.3)	0 (0.0)	1 (0.6)	
18.5-24.0	81 (26.9)	45 (33.6)	36 (21.6)	
25.0-29.9	154 (51.2)	62 (46.3)	92 (55.1)	
≥30	65 (21.6)	27 (20.1)	38 (22.8)	
Comorbidities				
Hypertension	184 (50.3)	75 (51.0)	109 (49.8)	0.815
Heart disease	66 (18.0)	24 (16.3)	42 (19.2)	0.487
Stroke	12 (3.3)	4 (2.7)	8 (3.7)	0.624
Diabetes	68 (18.6)	26 (17.7)	42 (19.2)	0.719
Lung disease	35 (9.6)	10 (6.8)	25 11.4)	0.141
Psychiatric disorder	21 (5.7)	6 (4.1)	15 (6.8)	0.264
Nervous system disorder	8 (2.2)	1 (0.7)	7 (3.2)	0.107

COVID-19, coronavirus disease 2019.

a >20 g/day for men aged 18-64 years and >10 g/day for men aged ≥65.

b At least 150 minutes of physical activity weekly (minutes of moderate physical activity + 2 × minutes of vigorous physical activity).

Mean MoCA scores increased from baseline to the 1-year evaluation [mean (SD): 22.3 (3.7) versus 22.8 (3.8), respectively; *P* < 0.001), but this variation, when the 1-year evaluation was performed after the onset of COVID-19 pandemic, was not statistically significant.

Table 3 presents the mean difference in MoCA *t*-scores from baseline to the 1-year evaluation according to prostate cancer treatment. Only the group treated with ADT and chemotherapy, and those who underwent radical prostatectomy (without adjuvant radiotherapy) had a statistically significant increase in mean *t*-scores over time [mean difference of MoCA *t*-score at 1 year minus MoCA *t*-score at baseline (95% CI): 7.59 (0.52-14.67) and 3.73 (1.10-6.37), respectively]. Participants treated with ADT only had a nonstatistically significant decrease and the remaining treatment groups had nonstatistically significant increases. The increase in scores was less pronounced after the COVID-19 pandemic.

**Table 3:** Mean difference in the MoCA t-scores, according to cancer treatments (t-score at 1 year minus t-score at baseline)

Treatments	All		Moment of the 1-year evaluation			
			Before COVID-19		After COVID-19	
	*N*	Difference in MoCA t-scores^a^, mean (95% CI)	*N*	Difference in MoCA t-scores^a^, mean (95% CI)	*N*	Difference in MoCA t-scores^a^, mean (95% CI)
Active surveillance	18	0.601 (−3.760 to 4.962)	1	−17.778	17	1.682 (−2.279 to 5.643)
Brachytherapy	52	1.333 (−1.639 to 4.305)	22	2.359 (−1.623 to 6.341)	30	0.581 (−3.847 to 5.008)
RT	38	1.739 (−1.426 to 4.904)	12	3.996 (−2.705 to 10.698)	26	0.698 (−3.020 to 4.415)
RP	59	**3.731 (1.097 to 6.366)**	**25**	**4.211 (0.507 to 7.915)**	34	3.379 (−0.454 to 7.212)
RT + ADT 6 months	35	1.649 (−2.578 to 5.555)	8	4.319 (−6.816 to 15.454)	27	0.857 (−3.449 to 5.164)
RT + ADT 24 months^b^	90	1.233 (−0.775 to 3.241)	42	2.866 (−0.004 to 5.736)	48	−0.195 (−3.034 to 2.643)
ADT, incident PCa	22	−0.033 (−4.344 to 4.278)	12	1.582 (−2.920 to 6.084)	10	−1.971 (−10.778 to 6.836)
ADT + chemotherapy	12	**7.591 (0.516 to 14.667)**	5	7.651 (−0.685 to 15.986)	7	7.549 (−5.442 to 20.540)
ADT, recurrent PCa	25	0.249 (−4.939 to 5.436)	13	0.814 (−7.453 to 9.081)	12	−0.364 (−7.873 to 7.145)
RT + palliative ADT	1	10.490	0	—	1	10.490
RP + RT	13	0.877 (−4.823 to 6.576)	6	−1.159 (−10.443 to 8.124)	7	2.622 (−6.854 to 12.099)
RP + ADT	1	−1.748	1	−1.748	0	—
Total	366	1.738 (0.687 to 2.794)	147	**2.623 (1.019 to 4.227)**	219	1.143 (−0.260 to 2.547)

Results in bold correspond to statistically significant variations.

ADT, androgen-deprivation therapy; CI, confidence interval; COVID-19, coronavirus disease 2019; MoCA, Montreal Cognitive Assessment; PCa, prostate cancer; RP, radical prostatectomy; RT, radiotherapy; SD, standard deviation.

a Based on the mean and SD of age- and education-specific norms, ^20^ MoCA *z*-scores and *t*-scores were computed based on the formula (*z*-score × 10) + 50, to obtain a more intelligible score, so that most values are positive and vary from 0 to 100.

b Participants were proposed for 24 months of ADT and were still on ADT at the 1-year evaluation.

At baseline, 47 participants had cognitive impairment and of these, 51.6% scored within the normal MoCA range at the 1-year evaluation. Patients with cognitive decline presented a variation in MoCA scores that ranged from −9 to −4 points.

Table 4 presents the percentage of participants with cognitive decline and with incident cognitive impairment at the 1-year evaluation according to treatments received. None of the patients treated with prostatectomy or with radiotherapy only had cognitive decline. Patients with ADT as part of their treatments presented cognitive decline more often (range 7.8%-16.0%). There were 22 incident cases of cognitive impairment corresponding to a 1-year cumulative incidence of cognitive impairment of 6.9% (95% CI 4.3%-10.2%), which was higher after the COVID-19 pandemic (9.1% versus 3.7%; *P* = 0.057). Patients who received radiotherapy as an adjuvant treatment after radical prostatectomy had the highest 1-year cumulative incidence of cognitive impairment (15.4%), followed by those treated with radiotherapy combined with long-duration ADT (13.1%), and those treated with ADT for incident prostate cancer only (10.0%). None of the patients who received ADT and chemotherapy had incident cognitive impairment at 1 year.

**Table 4:** Cognitive outcomes at 1 year, according to prostate cancer treatment, before and after the COVID-19 pandemic

Treatments	Cognitive decline						Incident cognitive impairment					
	All		Moment of the 1-year evaluation				All		Moment of the 1-year evaluation			
			Before COVID-19		After COVID-19				Before COVID-19		After COVID-19	
	*N*	*n* (%)	*N*	*n* (%)	*N*	*n* (%)	*N* at risk	*n* (%)	*N* at risk	*n* (%)	*N* at risk	*n* (%)
Active surveillance	18	1 (5.6)	1	1 (100.0)	17	0 (0.0)	15	0 (0.0)	1	0 (0.0)	14	0 (0.0)
Brachytherapy	52	3 (5.8)	22	1 (4.5)	30	2 (6.7)	45	1 (2.2)	20	0 (0.0)	25	1 (4.0)
RT	38	0 (0.0)	12	0 (0.0)	26	0 (0.0)	34	0 (0.0)	11	0 (0.0)	23	0 (0.0)
RP	59	0 (0.0)	25	0 (0.0)	34	0 (0.0)	48	3 (6.3)	23	1 (4.3)	25	2 (8.0)
RT + ADT 6 months	35	3 (8.6)	8	1 (12.5)	27	2 (7.4)	28	2 (7.1)	6	0 (0.0)	22	2 (9.1)
RT + ADT 24 months^a^	90	7 (7.8)	42	1 (2.4)	48	6 (12.5)	84	11 (13.1)	40	1 (2.5)	44	10 (22.7)
ADT, incident PCa	22	3 (13.6)	12	1 (8.3)	10	2 (20.0)	20	2 (10.0)	11	1 (9.1)	9	1 (11.1)
ADT + chemotherapy	12	1 (8.3)	5	0 (0.0)	7	1 (14.3)	10	0 (0.0)	5	0 (0.0)	5	0 (0.0)
ADT, recurrent PCa	25	4 (16.0)	13	3 (23.1)	12	1 (8.3)	22	1 (4.5)	11	0 (0.0)	11	1 (9.1)
RT + palliative ADT	1	0 (0.0)	0	0 (0)	1	0 (0.0)	1	0 (0.0)	0	0 (0)	1	0 (0.0)
RP + RT	13	1 (7.7)	6	1 (16.7)	7	0 (0.0)	13	2 (15.4)	6	2 (33.3)	7	0 (0.0)
RP + ADT	1	1 (100.0)	1	1 (100.0)	0	0 (0)	1	0 (0.0)	1	0 (0.0)	0	0 (0.0)
Total	366	24 (6.6)	147	10 (6.8)	219	14 (6.4)	*321*	22 (6.9)	135	5 (3.7)	186	17 (9.1)

Differences between treatments: age (*P* < 0.001), education (*P* = 0.094), cognitive decline (*P* = 0.004), incident cognitive impairment (*P* = 0.285).

ADT, androgen-deprivation therapy; COVID-19, coronavirus disease 2019; PCa, prostate cancer; RP, radical prostatectomy; RT, radiotherapy.

a Participants were proposed for 24 months of ADT and were still on ADT at the 1-year evaluation.

**Figure 1:**
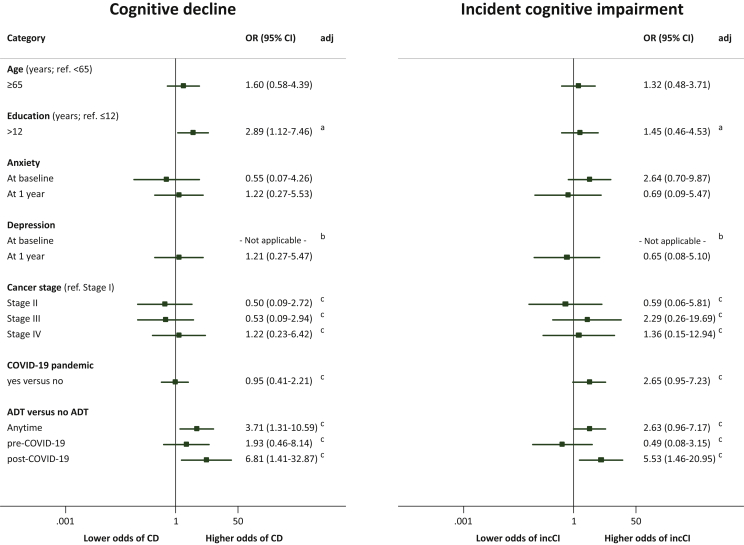
**Association of age, education, anxiety, and depression, and treatments with cognitive decline and with incident cognitive impairment.** 95% CI, 95% confidence interval; ADT, androgen-deprivation therapy; CD, cognitive decline defined as a variation in cognitive performance [Montreal Cognitive Assessment (MoCA) at 1 year minus MoCA at baseline] below 1.5 standard deviations of the variation in the whole cohort; COVID-19, coronavirus disease 2019; incCI, incident cognitive impairment defined as a score below age- and education-specific values from normative data at the 1-year evaluation in participants without cognitive impairment at baseline. ^a^Adjusted for age. ^b^None of the participants had the outcome (cognitive decline/incident cognitive impairment). ^c^Adjusted for age and education.

A higher educational level (>12 years) was associated with cognitive decline [age-adjusted OR (95% CI): 2.89 (1.12-7.46)]. Patients who underwent treatments including ADT had higher odds of cognitive decline compared with patients who were not treated with ADT [age and education aOR (95% CI): 3.71 (1.31-10.59)]. The moment of the 1-year assessment (before/after COVID-19) was not significantly associated with cognitive decline [aOR (95% CI): 0.95 (1.41-32.87)] and the interaction with ADT-based treatments was not statistically significant (*P* = 0.233), but the association between the COVID-19 pandemic and incident cognitive impairment was nearly statistically significant [aOR (95% CI): 2.65 (0.95-7.23)] and its interaction with ADT-based treatments was significant (*P* = 0.044). The association between ADT and incident cognitive impairment was only statistically significant after the pandemic [aOR (95% CI): 5.53 (1.46-20.95)]. Anxiety and depression symptoms were not associated with cognitive decline or incident cognitive impairment (Figure 1).

## Discussion

Overall, cognitive performance increased from baseline to the 1-year evaluation. Patients treated with ADT were more likely to have cognitive decline after 1 year of follow-up. The incidence of cognitive impairment was almost 7% and it was higher in patients treated with ADT, alone or with other treatments, but this effect was only observed when the 1-year assessment was conducted after the COVID-19 pandemic.

In the current study, mean MoCA scores increased over time, which was also observed in women with breast cancer during the first year after cancer diagnosis.^22^ This increase may reflect a practice effect, that is, an improvement due to becoming familiar with the testing procedures and the cognitive tasks but also due to a lower performance at baseline because of the overwhelming experience of a cancer diagnosis, and fear of treatments and prognosis, that may have dissipated after 1 year.^23^ Indeed, in the present study, borderline anxiety (a score ≥8 in the anxiety subscore of the HADS) was associated with MoCA scores at baseline, and patients proposed for radical prostatectomy had the lowest mean MoCA scores and the highest prevalence of borderline anxiety. However, this may not explain the low baseline MoCA scores in patients proposed for ADT and chemotherapy, as the prevalence of borderline anxiety was low in this group. Pain associated with bone metastases could explain the lower cognitive performance at baseline, although this assessment was usually performed after 3 weeks of antiandrogens for pain management and flare prevention. Pathological alterations due to cancer and the control of the disease after 1 year may explain low cognitive performance at baseline and improvement thereafter, respectively.

Cognitive decline, defined as having a variation in MoCA scores over time <1.5 SD of the variation in the cohort, was consistently more frequent in participants treated with ADT, regardless of the duration of ADT or associated treatments, and the incident or recurrent nature of the disease. This result supports the evidence from previous studies reporting an association of ADT with cognitive decline.^4^ In cross-sectional evaluations, a higher educational level has been associated with better cognitive performance.^24^ However, similar or higher rate of decline in some cognitive domains were reported in older adults with higher versus lower education.^25^ Moreover, among patients with incident Alzheimer’s disease, a more accelerated cognitive decline was reported in individuals with higher education.^26^ In the present study, patients with >12 years of education were more likely to belong to the group with the worst variation in cognitive scores over 1 year of follow-up, but no association with incident cognitive impairment was observed.

Most of the cases with cognitive decline (13/22) had high baseline MoCA score, which decreased at least 4 points, while remaining within the normal range for the specific age and education group. By contrast, most cases of incident cognitive impairment (also 13/22) had a decrease in MoCA scores between 1 and 3 points. Future assessments of the participants, as well as confirmation of cognitive impairment, with a battery of neuropsychological tests and a neurologist diagnosis are needed to refine these results, considering, in one hand, that a very high or low score at baseline or at 1 year may be due to chance only, being the variation observed a result of the phenomenon of regression to the mean, and, in the other hand, that the MoCA is a screening test.

In another longitudinal study of cognitive performance over a 5-year period in patients with breast cancer, the variation in MoCA scores in the first year of follow-up was a significant predictor of long-term cognitive decline.^22^ Although population- and cancer-specific differences may not allow to extrapolate the findings to the present study, the incidence of cognitive impairment at 1 year was similar to that observed among women with breast cancer 1 year after cancer diagnosis and using the MoCA (8.1%).^27^ These are two different populations of patients with cancer, regarding not only sex but also age and treatments. To our knowledge, there are no studies reporting the incidence of cognitive impairment in patients with prostate cancer.^11^ Patients treated with ADT were more likely to develop cognitive impairment, a consistent observation considering ADT alone or with radiotherapy, although none of the participants treated with ADT and chemotherapy had incident cognitive impairment. Patients proposed for chemotherapy were younger than those with ADT, which could explain this difference in the cognitive impairment incidence, as well as unmeasured factors related to overall health and lifestyle. In addition, docetaxel may not have deleterious effects in cognitive function as other drugs or combinations of drugs used in other cancers. Finally, this null result should be interpreted considering that there was a small number of patients treated with this drug, precluding a definitive conclusion on the effect of docetaxel on cognitive function.

The first COVID-19 case in Portugal was reported on 2 March 2020, and the NEON-PC cohort evaluations were suspended from 9 March to 1 July 2020. The first general lockdown occurred from 22 March to 30 April 2020 and the second between 16 January and 15 March 2021, during which the general population was forbidden from using public spaces, and compulsory confinement was legally imposed, except for basic shopping necessities, health consultations and treatments, and going to work when working from home was not possible.^28^ Total confinement and restrictions to normal daily activities since March 2020 have caused many alterations in everyone’s life, with a decrease in physical activity and an increase in sedentary behaviours,^29^ and changes in eating patterns.^30^ Moreover, the reduction in contact with nature was associated with worse mental health,^31^ and sleep problems were frequent during the COVID-19 pandemic.^32^ ADT has been associated with a higher risk for weight gain and metabolic syndrome,^33^ depression,^34^ and sleep disturbances.^35^ These adverse effects of ADT are associated with cognitive dysfunction,^36^, ^37^, ^38^, ^39^, ^40^ acting as potential mediators of the effect of ADT on cognitive performance. We observed a negative effect of ADT on the incidence of cognitive impairment, but only after the COVID-19 pandemic, which may be explained by a worsening effect of the pandemic in the prevalence of metabolic syndrome, depression, and sleep problems among patients who received ADT.

### Strengths and limitations

This is the largest prospective study comparing cognitive decline in patients with prostate cancer treated with or without ADT, and the first to report cognitive impairment cumulative incidence in these patients. Although neuropsychological tests are considered the gold standard to assess cognitive performance,^41^ which and how many tests to include to assess which cognitive domains, and the criteria to define cognitive impairment have not yet been standardized. Moreover, neuropsychological assessment may not be feasible both in clinical practice and in research. Indeed, due to the long duration for the administration of the battery of tests (at least one hour), the availability of neuropsychologists to administer and score the tests, and the willingness of participants to perform such long sessions may compromise the execution of comprehensive neuropsychological evaluations. Even while using a cognitive test that may not detect subtle changes in cognitive performance, our results show that ADT is associated with the deterioration of overall cognitive function.

The 1-year follow-up may not have been sufficient to detect the association of ADT with incident cognitive impairment in patients with the two evaluations performed before the COVID-19 pandemic, and future evaluations of the cohort may detect the effect of cumulative exposure to this therapy.

Although this study was conducted in only one hospital, IPO-Porto receives patients from all over the country, though mostly from the North, and it is the largest cancer-dedicated public hospital in Portugal.

### Conclusion

Patients with prostate cancer treated with ADT are more likely to have a deterioration in cognitive performance 1 year after initiating treatment. Therefore cognitive assessment should be considered in the clinical follow-up protocols of these patients. Socioeconomic, lifestyle, and clinical characteristics should also be considered in-depth to identify the moderators of the association of ADT with cognitive performance, and studies with longer follow-up are needed to understand if the negative effect of ADT is reversible after treatment termination. The COVID-19 pandemic may have worsened the effect of ADT in the cognitive performance of patients with prostate cancer.
